# Microstructure and Mechanical Properties Evolution of the Al, C-Containing CoCrFeNiMn-Type High-Entropy Alloy during Cold Rolling

**DOI:** 10.3390/ma11010053

**Published:** 2017-12-29

**Authors:** Margarita Klimova, Nikita Stepanov, Dmitry Shaysultanov, Ruslan Chernichenko, Nikita Yurchenko, Vladimir Sanin, Sergey Zherebtsov

**Affiliations:** 1 Laboratory of Bulk Nanostructured Materials, Belgorod State University, Belgorod 308015, Russia; klimova_mv@bsu.edu.ru (M.K.); shaysultanov@bsu.edu.ru (D.S.); rus.chernichenko@mail.ru (R.C.); yurchenko_nikita@bsu.edu.ru (N.Y.); zherebtsov@bsu.edu.ru (S.Z.); 2 Institute of Structural Macrokinetics and Materials Science, Russian Academy of Sciences, Moscow 142432, Russian; svn@ism.ac.ru

**Keywords:** high-entropy alloys, microstructure evolution, twinning, mechanical properties, strengthening mechanisms

## Abstract

The effect of cold rolling on the microstructure and mechanical properties of an Al- and C-containing CoCrFeNiMn-type high-entropy alloy was reported. The alloy with a chemical composition (at %) of (20–23) Co, Cr, Fe, and Ni; 8.82 Mn; 3.37 Al; and 0.69 C was produced by self-propagating high-temperature synthesis with subsequent induction. In the initial as-cast condition the alloy had an face centered cubic single-phase coarse-grained structure. Microstructure evolution was mostly associated with either planar dislocation glide at relatively low deformation during rolling (up to 20%) or deformation twinning and shear banding at higher strain. After 80% reduction, a heavily deformed twinned/subgrained structure was observed. A comparison with the equiatomic CoCrFeNiMn alloy revealed higher dislocation density at all stages of cold rolling and later onset of deformation twinning that was attributed to a stacking fault energy increase in the program alloy; this assumption was confirmed by calculations. In the initial as-cast condition the alloy had low yield strength of 210 MPa with yet very high uniform elongation of 74%. After 80% rolling, yield strength approached 1310 MPa while uniform elongation decreased to 1.3%. Substructure strengthening was found to be dominated at low rolling reductions (<40%), while grain (twin) boundary strengthening prevailed at higher strains.

## 1. Introduction

High-entropy alloys (HEAs) have recently emerged as new class of metallic materials with properties attractive for various structural and functional applications [1,2,3,4]. In particular, different HEAs demonstrate high strength and/or ductility at room and cryogenic temperatures, high specific strength at elevated temperatures, excellent fracture and impact toughness, wear resistance, etc. [5,6,7,8,9,10,11,12,13,14,15,16,17,18]. Producing HEAs with a balanced combination of properties needed for practical applications still remains a significant challenge, however.

Alloys based on the transition elements are the most studied type of HEAs [1]. One of the typical representatives of such HEAs is the equiatomic CoCrFeNiMn alloy [19,20,21]. This alloy has a single face centered cubic (fcc) phase structure thermodynamically stable at temperatures ≥900 °C [22,23,24]. It has very high ductility (70–80%) at both room and cryogenic temperatures, and record breaking fracture toughness under cryogenic conditions [5,9]. The encouraging properties of the alloy at cryogenic temperature were mostly attributed to pronounced deformation nano-twinning promoting high strain hardening capacity [5,9,25]. However, there are still different opinions on relative contributions of dislocation slip and twinning during plastic deformation at room temperature [9,26,27,28,29].

Apparently, mechanical properties of the CoCrFeNiMn alloy can be improved either by thermomechanical processing (resulting in a microstructure modification) or by alloying (changing the chemical composition). Both options have been explored by researchers already. For instance, it was found that cold rolling of the CoCrFeNiMn resulted in a considerable increase in strength with a simultaneous decrease in ductility [27]; however, microstructure refinement due to recrystallization can result in higher strength without a loss in ductility [9,30]. Doping with other elements (as well as changes in the concentrations of the principal elements) can be used either to enhance solid solution strengthening while maintaining a single phase solid solution structure and/or to cause the formation of second phases and to increase the strength via precipitation strengthening [10,31,32,33,34,35,36,37,38,39]. Both substitutional (an effective example is Al [38]) and interstitial (like C [31]) solid solution strengthening can be utilized.

However, modifications of the chemical composition of the fcc solid solution can also result in changes of different strengthening mechanisms activity. For example, intensive deformation twinning in the CoCrFeNiMn alloy was usually ascribed to a considerable decrease in the stacking fault energy (SFE) values to 20–30 mJ·m^−2^ [40,41]; an increase in the SFE values due to alloying can result in twinning suppression that inevitably would have a pronounced effect on the mechanical behavior of the alloys. It has been established earlier that Al and C in austenitic (fcc) steels are among the elements that strongly increase the SFE, thereby promoting dislocation slip and suppressing twinning [42,43,44]. However, there are contradicting reports on the effect of carbon doping on deformation mechanisms operating in the Co-Cr-Fe-Ni-Mn system high entropy alloys [12,32,33,45]. The effect of Al is even much less studied, however, deformation twinning was reported for the Al0.1CoCrFeNi alloy, nominally containing ~2.4 at % of Al [46,47]. Therefore, additional studies on the effects of alloying on deformation mechanisms of the CoCrFeNiMn-type alloys are required.

To explore operating deformation mechanisms, microstructure evolution during rolling and tensile properties of the CoCrFeNiMn-type HEA, containing ~3.4 at % of Al and ~0.7 at % of C was studied in detail. The following main aims were pursued: (i) to establish active deformation mechanisms at different stages of plastic deformation; and (ii) to estimate, quantitatively, the contributions of different strengthening mechanisms during cold working. Only a limited number of thorough investigations of this kind on HEAs have been made so far [27,34,48,49,50].

## 2. Results

### 2.1. Microstructure of the Al-, C-Containing CoCrFeNiMn-Type Alloy in the as-Cast Condition

**Figure 1:**
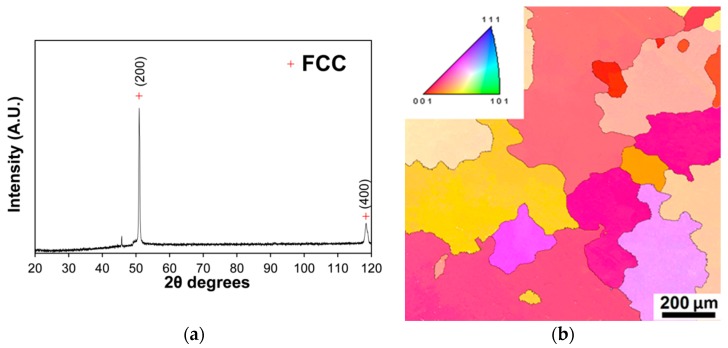
Structure of the Al-, C-containing CoCrFeNiMn-type alloy in the as-cast condition: (**a**) XRD pattern; and (**b**) EBSD IPF map.

Figure 1 illustrates the microstructure of the Al-, C-containing CoCrFeNiMn-type alloy in the initial as-cast condition. Both the X-ray diffraction (XRD) pattern (Figure 1a) and electron backscattered diffraction (EBSD) inverse pole figure (IPF) map (Figure 1b) demonstrated the presence of a single phase with the fcc lattice. According to the XRD results, the fcc lattice parameter a was 3.588 nm. In addition, both the XRD and EBSD data revealed strong crystallographic texture, typical for the cast materials. The alloy had a coarse structure with a grain size of 250–400 μm. Grains of irregular shape were often surrounded by curved boundaries. Additional investigations by scanning electron microscopy (SEM) and transmission electron microscopy (TEM) (not shown) have also not revealed the presence of any second phases. The chemical composition of the grains determined by SEM-based energy-dispersive X-ray spectroscopy (EDX) strictly corresponded to the composition of the alloy (Table 1).

### 2.2. Microstructure Evolution of the Al-, C-Containing CoCrFeNiMn-Type Alloy during Cold Rolling

**Figure 2:**
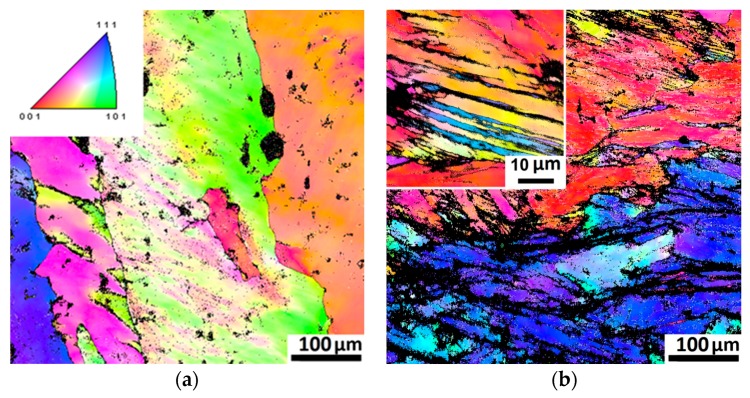

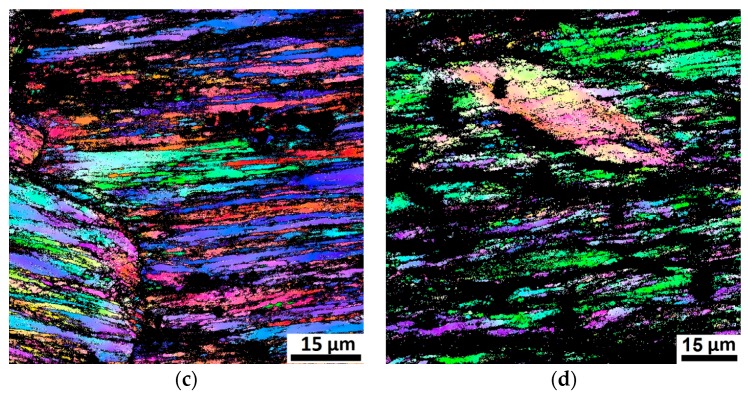
EBSD IPF maps of the Al-, C-containing CoCrFeNiMn-type alloy after cold rolling with different thickness reduction: (**a**) 20%; (**b**) 40%; (**c**) 60%; and (**d**) 80%. Rolling direction is aligned with the horizontal axis.

EBSD IPF maps (Figure 2) shows microstructure evolution of the Al-, C-containing CoCrFeNiMn-type alloy during cold rolling. Noticeable changes in the microstructure were observed only for ε = 20% (Figure 2a). After 20% thickness reduction the development of a banded substructure inside initial grains was observed (Figure 2a). With an increase in rolling strain, the initial grains elongated towards the rolling direction (Figure 2b). The grains were subdivided by deformation bands (dark areas in Figure 2b). The development of deformation twinning in some grains was also observed (higher magnification insert in Figure 2b). At yet higher rolling reduction (60%) more extensive development of twinning was found (Figure 2c). However, even after rolling to the highest strain of 80% some areas with only dislocation substructure were observed (Figure 2d). Twin boundaries and deformation bands aligned along the rolling direction with an increase in rolling reduction.

**Figure 3:**
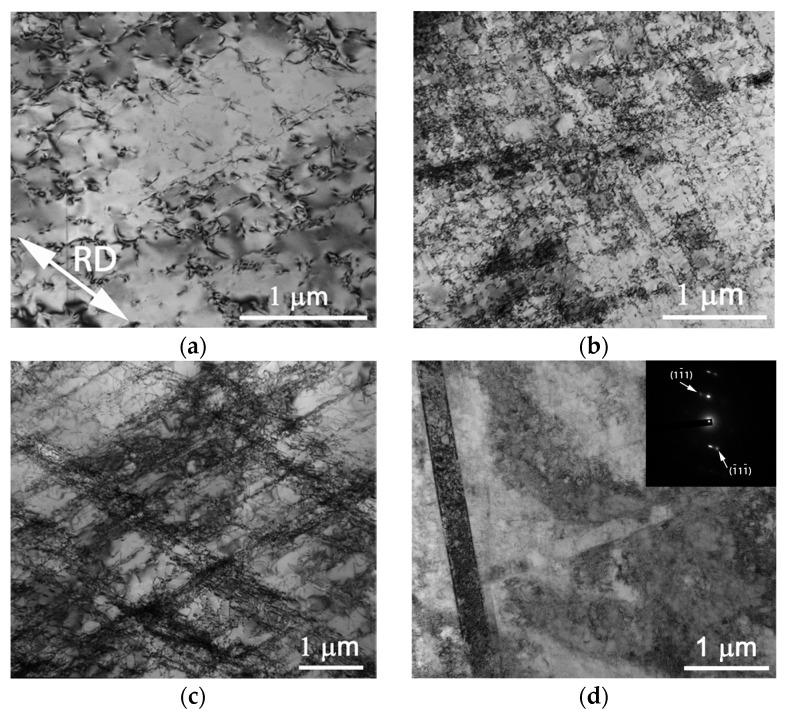

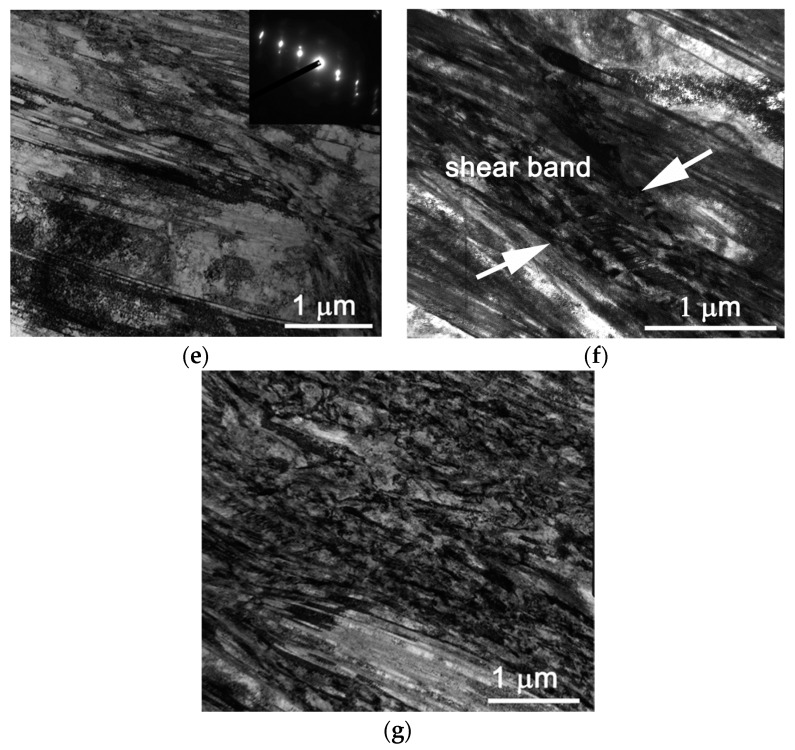
TEM bright-field images of the microstructure of the Al-, C-containing CoCrFeNiMn-type alloy after rolling with different thickness reduction: (**a**) 5%; (**b**) 10%; (**c**,**d**) 20%; (**e**) 40%; (**f**) 60%; and (**g**) 80%. Rolling direction (RD) is identified with the arrow in Figure 3a.

TEM investigations revealed additional insights into the evolution of the microstructure of the Al-, C-containing CoCrFeNiMn-type alloy (Figure 3). Pronounced dislocation activity was observed at the initial stages of deformation (Figure 3a). Dislocation slip was planar (Figure 3b); intersection of slip bands was found at higher strains (Figure 3c). Some individual, relatively thick twins started to appear at the same time (Figure 3d). High dislocation density inside twins should be noted. At 40% of thickness reduction intensive twinning was observed (Figure 3e). Deformation twins belonged to the (111) <112> family (twin/matrix misorientation of 60° around <111>) (insert on Figure 3e). The fraction of deformation twins obviously increased with further strain (Figure 3f). However, some areas comprised of only dislocation pile-ups and subboundaries were also observed. The formation of shear bands occurred concurrently (highlighted with the arrows in Figure 3f). As the result after 80% rolling inhomogeneous microstructure composed of twinned and subgrained areas was formed (Figure 3g).

**Figure 4:**
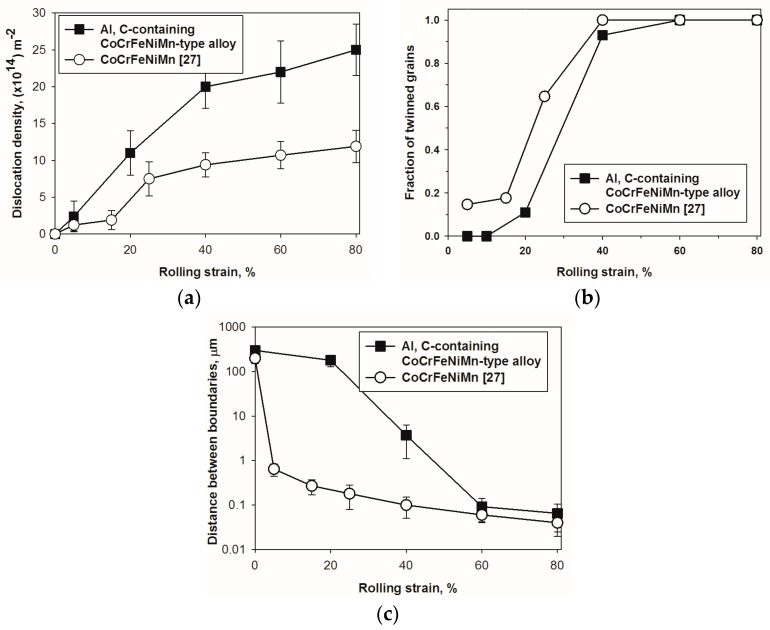
Dependence of (**a**) dislocation density; (**b**) fraction of twinned grains; and (**c**) distance between boundaries of the Al-, C-containing CoCrFeNiMn-type alloy on rolling strain. Data for the equiatomic CoCrFeNiMn alloy [27] is shown for comparison.

Quantitative analysis (Figure 4) of microstructure evolution of the Al-, C-containing CoCrFeNiMn-type alloy generally confirmed the results obtained by EBSD and TEM. Dislocation density increased relatively quickly at the initial stages of strain; from ~1 × 10^11^ m^−^^2^ in the initial condition to ~2 × 10^15^ m^−^^2^ after 40% strain (Figure 4a). At further rolling the increase in the dislocation density become much slower. Note that in the reference CoCrFeNiMn the overall dependence of dislocation density on rolling strain was similar, however, the “saturation” stage was reached already after 25% reduction, and dislocation densities at the same or comparable rolling strains were ca. two times lower than those in the present alloy. On the other hand, twinning obviously occurred much earlier in the reference CoCrFeNiMn alloy in comparison with the Al-, C-containing CoCrFeNiMn-type alloy: The first twins were detected only after 20% rolling reduction (Figure 4b, see also Figure 3d). However, twinning was developed extremely fast and already after 40% strain almost all grains were twinned (see also Figure 2b). Note that the development of twinning resulted in a sharp decrease in spacing between boundaries in the Al-, C-containing CoCrFeNiMn-type alloy at strains ≥ 40% (Figure 4c). The inter-twin distances attained values of about 0.06 μm at ε = 80%. In the reference CoCrFeNiMn alloy the spacing between boundaries decreased much faster due to earlier onset of twinning.

### 2.3. Mechanical Properties of the Al-, C-Containing CoCrFeNiMn-Type Alloy

**Figure 5:**
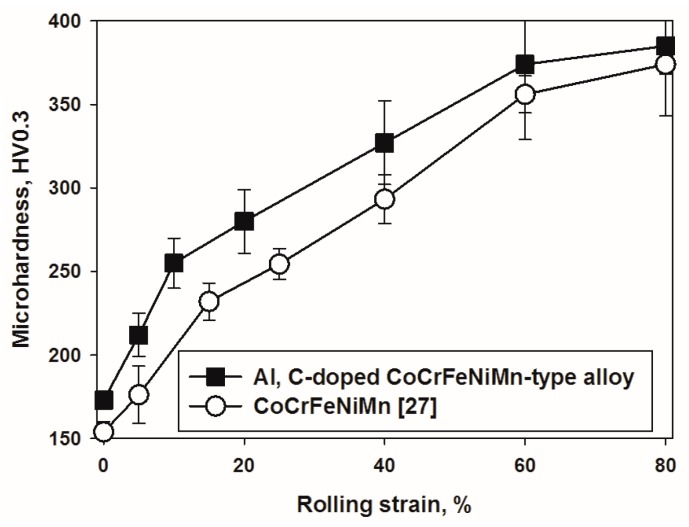
The dependence of microhardness of the Al-, C-containing CoCrFeNiMn-type alloy on rolling strain. Data for the equiatomic CoCrFeNiMn alloy [27] is shown for the comparison.

Figure 5 shows microhardness evolution of the Al-, C-containing CoCrFeNiMn-type alloy during rolling. In the initial as-cast condition the hardness of the Al-, C-containing alloy was 173 HV_0.__3_. During cold rolling the hardness of the alloy increased rapidly to 280 HV_0.3_ after ε = 20% and then, a bit slower, to 384 HV_0.3_ after ε = 60%. A further increase in strain to 80% did not result in noticeable changes in microhadness. The microhardness evolution of the Al, C-containing CoCrFeNiMn-type alloy is similar to that of the Al-, C-free CoCrFeNiMn alloy, yet the microhadness values of the present alloy were ≈20–40 HV_0.3_ higher than that of the reference alloy.

**Figure 6:**
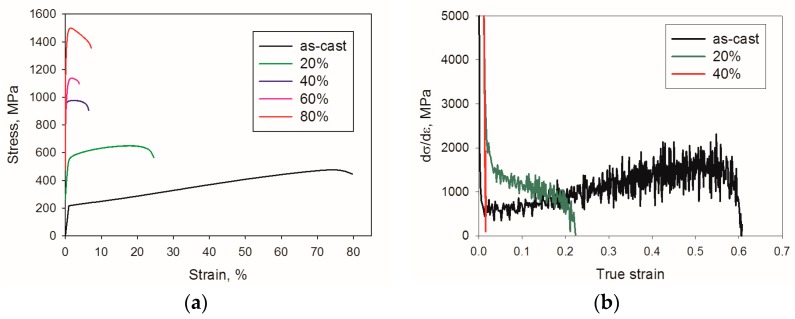
(**a**) Tensile stress-strain curves and (**b**) strain hardening curves of the Al-, C-containing CoCrFeNiMn-type alloy in the as-cast condition and after cold rolling with different thickness reductions.

More detailed understanding of the effect of cold rolling on mechanical properties of the Al-, C-containing CoCrFeNiMn-type alloy can be obtained via tensile tests. The representative stress-strain curves are shown in Figure 6, and the mechanical properties, namely yield strength (σ_0.2_), ultimate tensile strength (σ_UTS_), uniform elongation (ε_u_), and elongation to fracture (ε_f_), are summarized in Table 2. The as-cast alloy demonstrated low yield strength of 210 MPa with a pronounced hardening stage resulting in very high uniform elongation of 74%. The ultimate tensile strength and elongation to fracture of the cast alloy were 455 MPa and 80%, respectively. Rolling to relatively small thickness reduction (ε = 20%) resulted in pronounced hardening; the yield strength and ultimate tensile strength increased to 545 MPa and 650 MPa, respectively. However, the work hardening capacity of the alloy became lower with strain. Thus, the uniform elongation and elongation to fracture decreased to 18% and 25%, respectively. A further increase in rolling reduction to 40% increased strength and pronouncedly decreased ductility. The ultimate tensile strength of the alloy cold rolled to ε = 40% was found to be 980 MPa, while the uniform and total elongations were 3.7% and 7%, respectively. Rolling to higher strains resulted in even more pronounced hardening. For example, the ultimate tensile strength of the alloy was 1140 MPa and 1500 MPa after rolling to 60% and 80% reduction, respectively. However, the strengthening was associated with a significant decrease in ductility. The uniform elongation of the Al, C-containing CoCrFeNiMn-type alloy after rolling to 60% and 80% strain was 2.3% and 1.3%, respectively.

Strain hardening of the alloy in the initial as-cast condition (Figure 6b) was characterized by a reduction in hardening at the initial stage of deformation (to the true strain of 0.05) further increasing to the true strain of ~0.55, and then dropping to zero. The alloy after 20% rolling does not show any increase in the dσ/dε values during deformation, however, a lower rate of strain hardening decreasing can be seen in the interval of strains 0.05–0.2. All specimens of the alloy rolled to 40–80% (only one of them is shown in Figure 6b) demonstrate a sharp drop in strain hardening almost immediately after the beginning of deformation.

## 3. Discussion

The main features of the microstructure evolution of the Al-, C-containing CoCrFeNiMn-type high-entropy alloy during cold rolling comprised planar dislocation slip followed by intensive twinning and pronounced formation of shear bands (Figure 2, Figure 3 and Figure 4). The same mechanisms were found to operate in the reference equiatomic CoCrFeNiMn alloy [27], however, relative contributions of these mechanisms at different stages were pronouncedly different (Figure 4). Changes of deformation mechanisms in the fcc metals can be associated with the value of the stacking fault energy. However the exact calculation of the SFE value (which is a complex function of chemical composition of the alloy) is a very intricate problem. This value cannot be evaluate on the basis of already known data (for example the SFE for the equiatomic CoCrFeNiMn is known to be 20–30 mJ·m^−2^) since the composition of the program alloy differs from the classic equiatomic Cantor’s alloy both by the presence of Al and C and by concentrations of principal elements (Table 1).

A relatively simple approach to estimate the SFE of Fe-Mn steels was proposed in [51,52]. According to this approach, the SFE values can be calculated in accordance to the following formula:

(1) $$\gamma_{\mathrm{SFE}}=2\rho \Delta G_{\mathrm{hcp}-\mathrm{fcc}}+2\sigma$$

where γ_SFE_ is the SFE value, ∆G_hcp−fcc_ is the difference in Gibbs free energy between the hexagonal close packed (hcp) and fcc phases, ρ is the number of atoms per m^2^ in one atomic layer, and σ is the interphase energy between the fcc and hcp phases. Given the already-reported similarity in composition, structure, and mechanical behavior of the Co-Cr-Fe-Ni-Mn high-entropy alloys and high-Mn twinning-induced plasticity (TWIP) steels [27,53,54], Equation (1) was adapted in the present work to evaluate the difference in the SFEs between the reference Cantor’s CoCrFeNiMn alloy and the program Al-C-containing alloy. For this purpose, the ∆G_hcp−fcc_ values were calculated in both alloys using CALPHAD approach and commercial Thermo-Calc software (v. 2017a, Thermo-Calc Software, Stockholm, Sweden) and TCHEA2 database. Actual (Table 1) chemical composition of the Al-, C-containing alloy and nominal values (i.e., 20 at % of each element) of the CoCrFeNiMn alloys were used. The calculations were performed for room temperature (20 °C). The obtained results were values of ∆G_hcp−fcc_ = 0.72 kJ/mol for the reference CoCrFeNiMn alloy and 0.98 kJ/mol for the studied Al-, C-containing alloy. Although further exact calculations of the SFE were complicated due to the absence of other input values for Equation (1), the comparison of the calculated ∆G_hcp−fcc_ values clearly suggests that SFE of the Al-, C-containing alloy is higher than its CoCrFeNiMn equiatomic counterpart.

The increase in the SFE energy can perfectly explain the retardation of twinning in the Al-, C-containing CoCrFeNiMn-type alloy (Figure 4a). Greater SFE resulted in higher stresses required for twinning initiation; thus, the onset of deformation in the program alloy was generally associated with slip promoting a more intensive increase in dislocation density. The activation of twinning occurs when the flow stress of the material increases due to strain hardening. Since the strain hardening behavior of both alloys (Figure 5) is more or less similar, the required stress level reached at later stages of deformation in the Al, C-containing alloy in comparison with the equiatomic counterpart. Due to mainly planar slip (Figure 2b,d), recovery is limited and therefore dislocation density in the Al, C-containing alloy remains to be very high. Note that strongly planar slip was also found in high-Mn steels with higher SFE than the TWIP steels (the-called low-density steels) [55,56].

Microstructure evolution during rolling obviously impacts the mechanical properties (Figure 5 and Figure 6, Table 2). Both an increase in dislocation density and decrease in boundary spacing, mostly due to mechanical twinning (Figure 4a,c, respectively) can contribute to the strengthening of the alloy during deformation. The overall strength of the alloy can be expressed as:

(2) $$\sigma =\sigma_{0}+\sigma_{\rho }+\sigma_{H-P}$$

where σ_0_ denotes the friction stress, σ_ρ_ is the substructure strengthening, expressed as:

(3) $$\sigma_{\rho }=M\alpha \mathrm{Gb}\sqrt{\rho }$$

and σ_H−P_ is the Hall-Petch strengthening:

(4) σH−P=Kyd−12

In these equations M is the Taylor factor, α is a constant, G is the shear modulus, b is the Burgers vector, and ρ is the dislocation density; K_y_ is the Hall-Petch coefficient and d is the grain size (boundary spacing).

**Figure 7:**
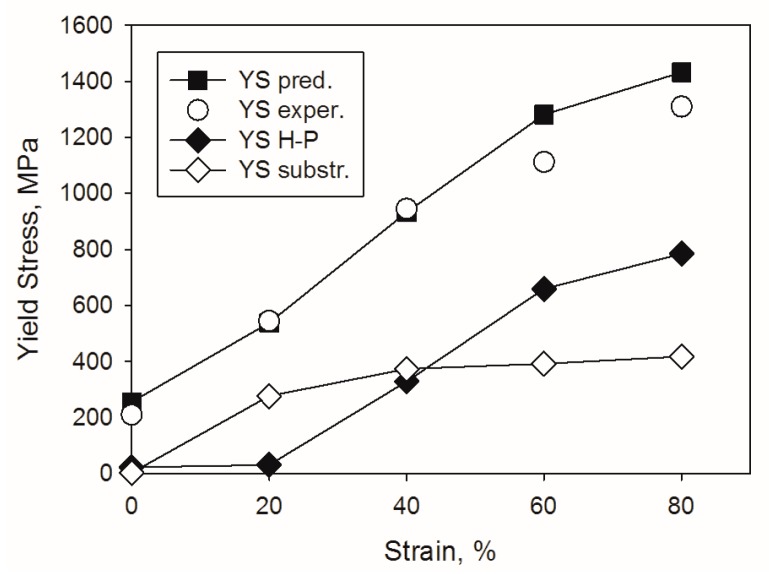
Contribution of different strengthening mechanisms to the overall strength of the Al-, C-containing CoCrFeNiMn-type alloy during rolling.

In the present work the following parameters were used: M = 2, α = 0.2, G = 80 GPа [57], b = 2.58 × 10^−^^10^ m [27]. The value of σ_0_ = 210 MPa was used in accordance with the experimental yield strength of the alloy in the initial coarse-grained condition (Table 2). The value of the Hall-Petch coefficient was experimentally determined as K_y_ = 0.4 MPa·m^−^^1/2^ for arbitrary grain boundaries. Twice lower K_y_ of 0.2 MPa·m^−^^1/2^ was used for twin boundaries (rolling reduction ≥40%) in accordance with the results of [58]. The values calculated using the Equations (2)–(4) are shown in Figure 7. The ρ and d values for the calculations were taken from Figure 4a,c, respectively.

The comparison between the experimental and the calculated (Equation (2)) values demonstrates a very good fit, especially for strains ε ≤ 40% (Figure 7). More detailed analysis shows that at ε = 20% reduction, the strength increases mostly due to substructure hardening while Hall-Petch strengthening is negligible. However, a further increase in strain does not result in a pronounced increase in substructure strengthening due to the saturation of dislocation density at strains ε ≥ 40% (Figure 4a). At the same time, grain boundary (Hall-Petch) strengthening grows continuously at ε ≥ 20% as a result of intensive twinning (Figure 4b). In general, the relative efficiency of the strengthening mechanisms operating in the Al-, C-containing CoCrFeNiMn-type alloy is in good agreement with microstructural observations (Figure 2, Figure 3 and Figure 4). The main deformation mode at the first stage of rolling was dislocation slip. The contributions of substructure and grain boundary strengthening became nearly equal at rolling reduction of 40%. During further deformation twinning makes the main contribution to deformation and Hall-Petch strengthening dominates due to the presence a large number of twin boundaries. Late development of deformation twinning resulted in rather specific shape of the strain hardening curve with increasing till the very end of deformation (Figure 6b). This result is quite similar to those obtained earlier for some TWIP steels [59] in which twinning proceeded during the greatest part of deformation. However, in such twinning alloys as commercially-pure titanium or brass, the stage of twinning-induced strain hardening growth is quite short and observed in the beginning of deformation [60]. Even small prestraining (in our case 20% rolling, Figure 6a, see also [59]) suppresses the effect of twinning on strain hardening, thereby considerably decreasing the ductility of the alloy.

It should be noted that in the equiatomic CoCrFeNiMn alloy dominant role of grain boundary strengthening within the whole (5–80%) strain range was established [27]. This is most possibly associated with the already-discussed larger propensity of the equiatomic alloy to twinning.

## 4. Materials and Methods

The initial Al-, C-containing CoCrFeNiMn-type alloy was produced by self-propagating high-temperature synthesis (SHS). Mixture of powders (oxides of the target elements (NiO, Cr_2_O_3_, Co_3_O_4_, Fe_2_O_3_, MnO_2_), pure carbon (C), and Al as the metal reducer) was used as a starting material. The weight of the initial mixture for combustion was 1200 g. Combustion was carried out in graphite molds 80 mm in diameter. Previous studies have demonstrated that the SHS process carried out under high-gravity conditions allows the best separation of the target product (ingot) from the slag (Al_2_O_3_) and convective mixing of all alloy components, which becomes especially important with an increased number of components and their concentration of components in the alloy. Therefore, the synthesis of the alloy was carried out in a centrifugal SHS setup. The SHS-produced HEA was 600 ± 10 g in weight and looked like a cast product. However, it contained numerous pores. Thus, the SHS-produced alloy was remelted using an induction furnace in vacuum and cast into an ingot measuring ~40 mm in diameter and ~80 mm in length. This was used as the starting condition. The chemical composition (metallic elements were determined by scanning electron microscope (SEM)-based energy-dispersive X-ray spectroscopy (EDX) scan using an FEI Nova NanoSEM 450 (ThermoFisher Scientific, Hillsboro, OR, USA) over the large area (~1 × 1 mm), carbon was measured by a Leco analyzer) is shown in Table 1.

Slabs with a thickness of 5 mm were cut from the ingot of the alloy by an electric discharge machine for further rolling. The slabs were rolled unidirectionally in a few passes at room temperature to a final thickness strain of 80%. Samples with intermediate thickness strains of 5%, 10%, 20%, 40%, and 60% were also produced. The reductions per pass were 5–10%. The rolling strain was calculated from the thickness of produced specimens.

The structure of the alloy was studied using X-ray diffraction (XRD) analysis, transmission electron microscopy (TEM), and electron backscattered diffraction (EBSD) analysis. The XRD analysis was performed using a RIGAKU diffractometer (Rigaku Corporation, Tokyo, Japan) and Cu Ka radiation.

Microstructural investigations were carried out in the plane perpendicular to the transversal direction. EBSD was conducted in an FEI Nova NanoSEM 450 field-emission-gun scanning electron microscope (FEG-SEM) (ThermoFisher Scientific, Hillsboro, OR, USA) equipped with a Hikari EBSD detector (EDAX Inc., Mahwah, NJ, USA) and TSL OIM™ system version 6.0 (EDAX Inc., Mahwah, NJ, USA). The samples for EBSD analysis were prepared by careful mechanical polishing. The border between low-angle boundaries (LABs, shown with white lines on presented inverse pole figure (IPF) maps) and high-angle boundaries (HABs, shown with black lines) was assumed to be 15°. Misorientations below 2° were not taken into consideration. The points with the confidence index (CI) below 0.1 were excluded from the analysis and were depicted as black dots on the presented IPF maps.

TEM investigations were performed using a JEOL JEM-2100 microscope (JEOL Ltd., Tokyo, Japan) with an accelerating voltage of 200 kV. The samples for the TEM analysis were prepared by conventional twin-jet electro-polishing of foils mechanically pre-thinned to 100 μm, in a mixture of 90% CH_3_COOH and 10% HClO_4_ at 30 V potential at room temperature. The dislocation density was estimated by counting the individual dislocations in the grains/subgrains interiors per unit area using TEM images.

To determine the post-rolling mechanical properties, tension tests were conducted at room temperature. For this purpose, dog-bone-shaped flat specimens with gauge dimensions of 5 mm length × 3 mm width × 1 mm thickness were machined and pulled to fracture at an initial strain rate of 10^−3^ s^−1^ using an Instron 5882 test machine (Instron, Norwood, MA, USA). At least two specimens were tested for each condition. Elongation to fracture was determined by measurements of spacing between marks designating the gauge length before and after the test. The microhardness of the rolled specimens was examined using Vickers microhardness (Instron, Norwood, MA, USA) testing with a load of 0.3 kg. At least 20 individual measurements per condition were made and the mean values are presented.

## 5. Conclusions

In the present work, the microstructure and mechanical properties evolution of the Al-, C-containing CoCrFeNiMn-type high-entropy alloy during cold rolling was studied. The following conclusions were drawn:

(1) The alloy produced by self-propagating high-temperature synthesis and further induction remelting was composed of ≈20–23 at % of Co, Cr, Fe, and Ni, 8.82 at % of Mn, 3.37 at % of Al, and 0.69 at % of C. The as-cast alloy had a coarse-grained single fcc phase structure with a grain size of 250–400 μm. The alloy had low yield strength of 210 MPa, but demonstrated a high uniform elongation of 74%.
(2) At the initial stages of rolling (thickness reduction <40%) mostly planar dislocation glide took place. First deformation twins appeared at a rolling reduction of 20%, and at 40% the strain of almost each grain contained at least one deformation twin. Formation of deformation twins resulted in a strong reduction of boundary spacing.
(3) A comparison with the equiatomic CoCrFeNiMn alloy demonstrated that dislocation density was ~2 times higher in the investigated Al-, C-containing alloy while twinning has initiated at later stages of deformation (20% reduction vs. 5% in the equiatomic alloy). The changes in deformation mechanisms were attributed to an increase in the stacking fault energy, which was confirmed by the Thermo-Calc estimation of the ∆G_hcp−fcc_ values for the alloys.
(4) Rolling resulted in an increase in strength and a decrease in ductility of the alloy. For instance, the yield strength of the alloy increased from 545 MPa to 1310 MPa when the rolling reduction changed from 20% to 80%, while the corresponding uniform elongation values were 18.0% and 1.3%, respectively.
(5) Analysis of the strengthening mechanisms has revealed that at rolling reductions <40% substructure strengthening prevailed, while at strains >40% grain (twin) boundary strengthening made the main contribution. At 40% reduction, both factors contributed equally.

## Figures and Tables

**Table 1:** Chemical composition of the Al-, C-containing CoCrFeNiMn-type alloy.

Concentration	Al	C	Co	Cr	Fe	Ni	Mn
at %	3.37	0.69	22.35	19.67	22.85	22.44	8.62
wt. %	1.65	0.15	23.92	18.57	23.17	23.92	8.64

**Table 2:** Tensile properties of the Al-, C-containing CoCrFeNiMn-type alloy in different conditions.

Condition	σ_0.2_, MPa	σ_UTS_, MPa	ε_u_, %	ε_f_, %
As-cast	210	455	74.0	80.0
20% rolling	545	650	18.0	25.0
40% rolling	945	980	3.7	7.0
60% rolling	1110	1140	2.3	5.4
80% rolling	1310	1500	1.3	6.5

## References

[1] Miracle D.B., Senkov O.N. (2017). A critical review of high entropy alloys and related concepts. Acta Mater..

[2] Zhang Y., Zuo T.T., Tang Z., Gao M.C., Dahmen K.A., Liaw P.K., Lu Z.P. (2014). Microstructures and properties of high-entropy alloys. Prog. Mater. Sci..

[3] Pickering E.J., Jones N.G. (2016). High-entropy alloys: A critical assessment of their founding principles and future prospects. Int. Mater. Rev..

[4] Tsai M.-H., Yeh J.-W. (2014). High-Entropy Alloys: A Critical Review. Mater. Res. Lett..

[5] Gludovatz B., Hohenwarter A., Catoor D., Chang E.H., George E.P., Ritchie R.O. (2014). A fracture-resistant high-entropy alloy for cryogenic applications. Science.

[6] Gludovatz B., Hohenwarter A., Thurston K.V.S., Bei H., Wu Z., George E.P., Ritchie R.O. (2016). Exceptional damage-tolerance of a medium-entropy alloy CrCoNi at cryogenic temperatures. Nat. Commun..

[7] Li D., Zhang Y. (2016). The ultrahigh charpy impact toughness of forged AlxCoCrFeNi high entropy alloys at room and cryogenic temperatures. Intermetallics.

[8] Chuang M.-H., Tsai M.-H., Wang W.-R., Lin S.-J., Yeh J.-W. (2011). Microstructure and wear behavior of AlxCo1.5CrFeNi1.5Tiy high-entropy alloys. Acta Mater..

[9] Otto F., Dlouhý A., Somsen C., Bei H., Eggeler G., George E.P. (2013). The influences of temperature and microstructure on the tensile properties of a CoCrFeMnNi high-entropy alloy. Acta Mater..

[10] He J.Y.Y., Wang H., Huang H.L.L., Xu X.D.D., Chen M.W.W., Wu Y., Liu X.J.J., Nieh T.G.G., An K., Lu Z.P.P. (2016). A precipitation-hardened high-entropy alloy with outstanding tensile properties. Acta Mater..

[11] Li Z., Pradeep K.G., Deng Y., Raabe D., Tasan C.C. (2016). Metastable high-entropy dual-phase alloys overcome the strength–ductility trade-off. Nature.

[12] Li Z., Tasan C.C., Springer H., Gault B., Raabe D. (2017). Interstitial atoms enable joint twinning and transformation induced plasticity in strong and ductile high-entropy alloys. Sci. Rep..

[13] Senkov O.N., Wilks G.B., Scott J.M., Miracle D.B. (2011). Mechanical properties of Nb25Mo25Ta25W25 and V20Nb20Mo20Ta20W20 refractory high entropy alloys. Intermetallics.

[14] Senkov O.N., Senkova S.V., Woodward C.F. (2014). Effect of aluminum on the microstructure and properties of two refractory high-entropy alloys. Acta Mater..

[15] Senkov O.N., Senkova S.V., Woodward C., Miracle D.B. (2013). Low-density, refractory multi-principal element alloys of the Cr–Nb–Ti–V–Zr system: Microstructure and phase analysis. Acta Mater..

[16] Stepanov N.D., Shaysultanov D.G., Salishchev G.A., Tikhonovsky M.A. (2015). Structure and mechanical properties of a light-weight AlNbTiV high entropy alloy. Mater. Lett..

[17] Yurchenko N.Y., Stepanov N.D., Shaysultanov D.G., Tikhonovsky M.A., Salishchev G.A. (2016). Effect of Al content on structure and mechanical properties of the AlxCrNbTiVZr (x = 0; 0.25; 0.5; 1) high-entropy alloys. Mater. Charact..

[18] Yurchenko N.Y., Stepanov N.D., Zherebtsov S.V., Tikhonovsky M.A., Salishchev G.A. (2017). Structure and mechanical properties of B2 ordered refractory AlNbTiVZrx (x = 0–1.5) high-entropy alloys. Mater. Sci. Eng. A.

[19] Cantor B., Chang I.T.H., Knight P., Vincent A.J.B. (2004). Microstructural development in equiatomic multicomponent alloys. Mater. Sci. Eng. A.

[20] Otto F., Yang Y., Bei H., George E.P.P. (2013). Relative effects of enthalpy and entropy on the phase stability of equiatomic high-entropy alloys. Acta Mater..

[21] Gludovatz B., George E.P., Ritchie R.O. (2015). Processing, Microstructure and Mechanical Properties of the CrMnFeCoNi High-Entropy Alloy. JOM.

[22] Otto F., Dlouhý A., Pradeep K.G., Kuběnová M., Raabe D., Eggeler G., George E.P. (2016). Decomposition of the single-phase high-entropy alloy CrMnFeCoNi after prolonged anneals at intermediate temperatures. Acta Mater..

[23] Schuh B., Mendez-Martin F., Völker B., George E.P.P., Clemens H., Pippan R., Hohenwarter A., Volker B., George E.P.P., Clemens H. (2015). Mechanical properties, microstructure and thermal stability of a nanocrystalline CoCrFeMnNi high-entropy alloy after severe plastic deformation. Acta Mater..

[24] Stepanov N.D.D., Shaysultanov D.G.G., Ozerov M.S.S., Zherebtsov S.V.V., Salishchev G.A.A. (2016). Second phase formation in the CoCrFeNiMn high entropy alloy after recrystallization annealing. Mater. Lett..

[25] Zhang Z., Mao M.M., Wang J., Gludovatz B., Zhang Z., Mao S.X., George E.P., Yu Q., Ritchie R.O. (2015). Nanoscale origins of the damage tolerance of the high-entropy alloy CrMnFeCoNi. Nat. Commun..

[26] Laplanche G., Kostka A., Horst O.M.M., Eggeler G., George E.P.P. (2016). Microstructure evolution and critical stress for twinning in the CrMnFeCoNi high-entropy alloy. Acta Mater..

[27] Stepanov N., Tikhonovsky M., Yurchenko N., Zyabkin D., Klimova M., Zherebtsov S., Efimov A., Salishchev G. (2015). Effect of cryo-deformation on structure and properties of CoCrFeNiMn high-entropy alloy. Intermetallics.

[28] Jang M.J., Ahn D.-H., Moon J., Bae J.W., Yim D., Yeh J.-W., Estrin Y., Kim H.S. (2017). Constitutive modeling of deformation behavior of high-entropy alloys with face-centered cubic crystal structure. Mater. Res. Lett..

[29] Joo S.-H., Kato H., Jang M.J., Moon J., Tsai C.W., Yeh J.W., Kim H.S. (2017). Tensile deformation behavior and deformation twinning of an equimolar CoCrFeMnNi high-entropy alloy. Mater. Sci. Eng. A.

[30] Sun S.J., Tian Y.Z., Lin H.R., Dong X.G., Wang Y.H., Zhang Z.J., Zhang Z.F. (2017). Enhanced strength and ductility of bulk CoCrFeMnNi high entropy alloy having fully recrystallized ultrafine-grained structure. Mater. Des..

[31] Wang Z., Baker I. (2016). Interstitial strengthening of a f.c.c. FeNiMnAlCr high entropy alloy. Mater. Lett..

[32] Wang Z., Baker I., Cai Z., Chen S., Poplawsky J.D., Guo W. (2016). The effect of interstitial carbon on the mechanical properties and dislocation substructure evolution in Fe40.4Ni11.3Mn34.8Al7.5Cr6 high entropy alloys. Acta Mater..

[33] Stepanov N.D., Yurchenko N.Y., Tikhonovsky M.A., Salishchev G.A. (2016). Effect of carbon content and annealing on structure and hardness of the CoCrFeNiMn-based high entropy alloys. J. Alloys Compd..

[34] Stepanov N.D., Shaysultanov D.G., Chernichenko R.S., Yurchenko N.Y., Zherebtsov S.V., Tikhonovsky M.A., Salishchev G.A. (2017). Effect of thermomechanical processing on microstructure and mechanical properties of the carbon-containing CoCrFeNiMn high entropy alloy. J. Alloys Compd..

[35] Liu C.M., Wang H.M., Zhang S.Q., Tang H.B., Zhang A.L. (2014). Microstructure and oxidation behavior of new refractory high entropy alloys. J. Alloys Compd..

[36] Gwalani B., Soni V., Lee M., Mantri S., Ren Y., Banerjee R. (2017). Optimizing the coupled effects of Hall-Petch and precipitation strengthening in a Al0.3CoCrFeNi high entropy alloy. Mater. Des..

[37] Liu W.H., Yang T., Liu C.T. (2017). Precipitation hardening in CoCrFeNi-based high entropy alloys. Mater. Chem. Phys..

[38] He J.Y.Y., Liu W.H.H., Wang H., Wu Y., Liu X.J.J., Nieh T.G.G., Lu Z.P.P. (2014). Effects of Al addition on structural evolution and tensile properties of the FeCoNiCrMn high-entropy alloy system. Acta Mater..

[39] Stepanov N.D., Shaysultanov D.G., Tikhonovsky M.A., Salishchev G.A. (2015). Tensile properties of the Cr-Fe-Ni-Mn non-equiatomic multicomponent alloys with different Cr contents. Mater. Des..

[40] Laplanche G., Kostka A., Reinhart C., Hunfeld J., Eggeler G., George E.P. (2017). Reasons for the superior mechanical properties of medium-entropy CrCoNi compared to high-entropy CrMnFeCoNi. Acta Mater..

[41] Liu S.F., Wu Y., Wang H.T., He J.Y., Liu J.B., Chen C.X., Liu X.J., Wang H., Lu Z.P. (2017). Stacking fault energy of face-centered-cubic high entropy alloys. Intermetallics.

[42] De Cooman B.C., Estrin Y., Kim S.K. (2018). Twinning-induced plasticity (TWIP) steels. Acta Mater..

[43] Saeed-Akbari A., Imlau J., Prahl U., Bleck W. (2009). Derivation and variation in composition-dependent stacking fault energy maps based on subregular solution model in high-manganese steels. Metall. Mater. Trans. A Phys. Metall. Mater. Sci..

[44] Schramm R.E., Reed R.P. (1975). Stacking fault energies of seven commercial austenitic stainless steels. Metall. Trans. A.

[45] Wu Z., Parish C.M., Bei H. (2015). Nano-twin mediated plasticity in carbon-containing FeNiCoCrMn high entropy alloys. J. Alloys Compd..

[46] Wu S.W., Wang G., Yi J., Jia Y.D., Hussain I., Zhai Q.J., Liaw P.K. (2017). Strong grain-size effect on deformation twinning of an Al0.1CoCrFeNi high-entropy alloy. Mater. Res. Lett..

[47] Yu P.F., Cheng H., Zhang L.J., Zhang H., Jing Q., Ma M.Z., Liaw P.K., Li G., Liu R.P. (2016). Effects of high pressure torsion on microstructures and properties of an Al0.1CoCrFeNi high-entropy alloy. Mater. Sci. Eng. A.

[48] Senkov O.N., Semiatin S.L. (2015). Microstructure and properties of a refractory high-entropy alloy after cold working. J. Alloys Compd..

[49] Hou J., Zhang M., Ma S., Liaw P.K., Zhang Y., Qiao J. (2017). Strengthening in Al0.25CoCrFeNi high-entropy alloys by cold rolling. Mater. Sci. Eng. A.

[50] Wang Z., Gao M.C., Ma S.G., Yang H.J., Wang Z.H., Ziomek-Moroz M., Qiao J.W. (2015). Effect of cold rolling on the microstructure and mechanical properties of Al0.25CoCrFe1.25Ni1.25 high-entropy alloy. Mater. Sci. Eng. A.

[51] Allain S., Chateau J.P., Bouaziz O. (2004). A physical model of the twinning-induced plasticity effect in a high manganese austenitic steel. Mater. Sci. Eng. A.

[52] Cotes S.M., Guillermet A.F., Sade M. (2004). Fcc/Hcp martensitic transformation in the Fe-Mn system: Part II. Driving force and thermodynamics of the nucleation process. Metall. Mater. Trans. A.

[53] Zaddach A.J., Scattergood R.O., Koch C.C. (2015). Tensile properties of low-stacking fault energy high-entropy alloys. Mater. Sci. Eng. A.

[54] Klimova M., Zherebtsov S., Stepanov N., Salishchev G., Haase C., Molodov D.A. (2017). Microstructure and texture evolution of a high manganese TWIP steel during cryo-rolling. Mater. Charact..

[55] Welsch E., Ponge D., Hafez Haghighat S.M., Sandlöbes S., Choi P., Herbig M., Zaefferer S., Raabe D. (2016). Strain hardening by dynamic slip band refinement in a high-Mn lightweight steel. Acta Mater..

[56] Haase C., Zehnder C., Ingendahl T., Bikar A., Tang F., Hallstedt B., Hu W., Bleck W., Molodov D.A. (2017). On the deformation behavior of κ-carbide-free and κ-carbide-containing high-Mn light-weight steel. Acta Mater..

[57] Wu Z., Bei H., Pharr G.M., George E.P. (2014). Temperature dependence of the mechanical properties of equiatomic solid solution alloys with face-centered cubic crystal structures. Acta Mater..

[58] Yanushkevich Z., Mogucheva A., Tikhonova M., Belyakov A., Kaibyshev R. (2011). Structural strengthening of an austenitic stainless steel subjected to warm-to-hot working. Mater. Charact..

[59] Kusakin P.S., Kaibyshev R.O. (2016). High-Mn twinning-induced plasticity steels: Microstructure and mechanical properties. Rev. Adv. Mater. Sci..

[60] Kalidindi S.R., Salem A.A., Doherty R.D. (2003). Role of deformation twinning on strain hardening in cubic and hexagonal polycrystalline metals. Adv. Eng. Mater..

